# T-Cell Lymphoblastic Lymphoma Arising in the Setting of Myeloid/Lymphoid Neoplasms with Eosinophilia: LMO2 Immunohistochemistry as a Potentially Useful Diagnostic Marker

**DOI:** 10.3390/cancers13123102

**Published:** 2021-06-21

**Authors:** Magda Zanelli, Giuseppe G. Loscocco, Elena Sabattini, Maurizio Zizzo, Francesca Sanguedolce, Luigi Panico, Daniela Fanni, Raffaella Santi, Cecilia Caprera, Cristiana Rossi, Alessandra Soriano, Alberto Cavazza, Alessandro Giunta, Cristina Mecucci, Alessandro M. Vannucchi, Stefano A. Pileri, Stefano Ascani

**Affiliations:** 1 Pathology Unit, Azienda Unità Sanitaria Locale—IRCCS di Reggio Emilia, 42123 Reggio Emilia, Italy; Magda.Zanelli@ausl.re.it (M.Z.); Alberto.Cavazza@ausl.re.it (A.C.); 2 Department of Experimental and Clinical Medicine, University of Florence, 50134 Florence, Italy; gloscocco@unifi.it (G.G.L.); a.vannucchi@unifi.it (A.M.V.); 3 Center of Research and Innovation of Myeloproliferative Neoplasms (CRIMM), Azienda Ospedaliero-Universitaria Careggi, 50139 Florence, Italy; 4 Haematopathology Unit, IRCCS Azienda Ospedaliero-Universitaria di Bologna, 40138 Bologna, Italy; elena.sabattini@aosp.bo.it; 5 Surgical Oncology Unit, Azienda Unità Sanitaria Locale—IRCCS di Reggio Emilia, 42123 Reggio Emilia, Italy; Alessandro.Giunta@ausl.re.it; 6 Clinical and Experimental Medicine PhD Program, University of Modena and Reggio Emilia, 41121 Modena, Italy; 7 Pathology Unit, Azienda Ospedaliero-Universitaria—Ospedali Riuniti di Foggia, 71122 Foggia, Italy; francesca.sanguedolce@unifg.it; 8 Pathology Unit Azienda Ospedaliera dei Colli Monaldi-Cotugno-CTO, P.O. Monaldi, 80131 Napoli, Italy; luigi.panico@ospedalideicolli.it; 9 Division of Pathology, Department of Medical Sciences and Public Health, University of Cagliari, 09042 Cagliari, Italy; danielafanni@aoucagliari.it; 10 Department of Pathology, Azienda Ospedaliero Universitaria Careggi, University of Florence, 50139 Florence, Italy; santir@aou-careggi.toscana.it; 11 Pathology Unit, Azienda Ospedaliera Santa Maria di Terni, University of Perugia, 05100 Terni, Italy; ceciliacaprera@libero.it (C.C.); s.ascani@aospterni.it (S.A.); 12 Pathology Unit, Azienda USL5, 19124 La Spezia, Italy; cristiana.rossi@asl5.liguria.it; 13 Department of Pathology, Case Western Reserve University, Cleveland, OH 44106, USA; Alessandra.Soriano@ausl.re.it; 14 Gastroenterology Unit, Azienda Unità Sanitaria Locale—IRCCS di Reggio Emilia, 42123 Reggio Emilia, Italy; 15 Haematology Unit, CREO, Azienda Ospedaliera di Perugia, University of Perugia, 06129 Perugia, Italy; cristina.mecucci@unipg.it; 16 Haematopathology Division, European Institute of Oncology—IEO IRCCS, 20141 Milan, Italy; stefano.pileri@ieo.it

**Keywords:** T-cell, lymphoblastic, lymphoma, eosinophilia, PDGFRA, PDGFRB, FGFR1, PCM1-JAK2

## Abstract

**Simple Summary:**

Rarely, T-lymphoblastic lymphoma (T-LBL) may develop in the setting of myeloid/lymphoid neoplasms with eosinophilia. Given important therapeutic implications, it is crucial to identify T-LBL arising in this particular context. LIM domain only 2 (LMO2) is known to be overexpressed in almost all sporadic T-LBL and not in immature TdT-positive T-cells in the thymus and in indolent T-lymphoblastic proliferations. We retrospectively evaluated the clinical, morphological, immunohistochemical and molecular features of 11 cases of T-LBL occurring in the setting of myeloid/lymphoid neoplasms with eosinophilia and investigated the immunohistochemical expression of LMO2 in this setting of T-LBL. Interestingly, 9/11 cases were LMO2 negative, with only 2 cases showing partial expression. In our study, we would suggest that LMO2 immunostaining, as part of the diagnostic panel for T-LBL, may represent a useful marker to identify T-LBL developing in the context of myeloid/lymphoid neoplasms with eosinophilia.

**Abstract:**

Background: Rarely, T-lymphoblastic lymphoma (T-LBL) may develop in the setting of myeloid/lymphoid neoplasms with eosinophilia (M/LNs-Eo), a group of diseases with gene fusion resulting in overexpression of an aberrant tyrosine kinase or cytokine receptor. The correct identification of this category has relevant therapeutic implications. LIM domain only 2 (LMO2) is overexpressed in most T-LBL, but not in immature TdT-positive T-cells in the thymus and in indolent T-lymphoblastic proliferations (iT-LBP). Methods and Results: We retrospectively evaluated 11 cases of T-LBL occurring in the context of M/LNs-Eo. Clinical, histological, immunohistochemical and molecular features were collected and LMO2 immunohistochemical staining was performed. The critical re-evaluation of these cases confirmed the diagnosis of T-LBL with morphological, immunohistochemical and molecular features consistent with T-LBL occurring in M/LNs-Eo. Interestingly, LMO2 immunohistochemical analysis was negative in 9/11 cases, whereas only 2 cases revealed a partial LMO2 expression with a moderate and low degree of intensity, respectively. Conclusions: LMO2 may represent a potentially useful marker to identify T-LBL developing in the context of M/LNs-Eo. In this setting, T-LBL shows LMO2 immunohistochemical profile overlapping with cortical thymocytes and iT-LBP, possibly reflecting different molecular patterns involved in the pathogenesis of T-LBL arising in the setting of M/LNs-Eo.

## 1. Introduction

T-lymphoblastic lymphoma (T-LBL) is an aggressive neoplasm of T-lymphoid precursors, which can rarely occur in the setting of M/LNs-Eo [1].

According to the current 2017 World Health Organization (WHO) classification, these disorders represent a distinct, but highly heterogeneous category, comprising cases with rearrangement of platelet-derived growth factor receptor (PDGFR)-alpha (PDGFRA), PDGFR-beta (PDGFRB) and fibroblast growth factor receptor 1 (FGFR1); a provisional entity identified by the presence of pericentriolar material 1-janus kinase 2 (PCM1-JAK2) rearrangement is also included [1,2,3,4]. A pluripotent stem cell is thought to be affected and disease presentation is very heterogeneous, including myeloproliferative neoplasms (MPN), myelodysplastic/myeloproliferative neoplasms (MDS/MPN), acute myeloid leukemia (AML), T- or B-LBL and mixed-phenotype acute leukemia [1,2,3,4]. The diagnosis of these entities requires the identification of the specific gene fusion, with cytogenetic and molecular tests, leading to overexpression of an aberrant tyrosine kinase or cytokine receptor [1,2,3,4]. PDGFRA and PDGFRB rearrangements may be cryptic, but fluorescence in situ hybridization (FISH) and/or RNA/DNA sequencing, either targeting or high-throughput next generation sequencing (NGS)-based are useful tools for diagnosis [1,2,3,4]. The identification of the underlying molecular abnormality is crucial, having important therapeutic implications, given the responsiveness of some of these disorders to tyrosine-kinase inhibitors (TKIs) [1,2,3,4,5]. The response to TKIs is different according to the fusion gene identified, as PDGFRA- and PDGFRB-rearranged cases are responsive, in the majority of cases, to imatinib, which represents the first line therapy, whereas FGFR1-rearranged cases do not respond to imatinib [3,5]. Although lymphoma-like aggressive chemotherapy and hematopoietic stem cell transplantation (HSCT) are considered the best therapeutic option for FGFR1-positive cases, prognosis is very poor in these cases. Clinical trials with new TKIs such as pemigatinib, a potent and selective inhibitor of FGFR family, are ongoing with promising preliminary results [6,7]. In patients with PCM1-JAK2 fusion gene, target therapy with JAK inhibitors such as ruxolitinib may offer a potential benefit [8].

LIM Domain Only 2 (LMO2), also known as rhombotin-like 1, is a highly evolutionary conserved protein involved in scaffolding of transcription factors (including GATA family) necessary for hematopoiesis and angiogenesis [9]. In recent years, it has been demonstrated that LMO2 protein is expressed in normal germinal centers (GCs) and germinal center (GC)-derived lymphomas [10]. Its overexpression has relevant prognostic implications in the context of diffuse large B-cell lymphoma (DLBCL), defining the GC molecular subgroup [11,12,13]. Moreover, LMO2 has been found to be overexpressed in the majority of T-LBL [14,15], but not in immature TdT-positive T-cells in the thymus and in indolent T-lymphoblastic proliferations (iT-LBP), as recently reported [16].

## 2. Results

The clinic-pathological features of T-LBL occurring in patients affected by M/LNs-Eo are summarized in Table 1. A detailed description of each case, including epidemiology, clinical presentation, treatment, outcome, histological, immunohistochemical and molecular findings, is presented in the Supplementary Material.

### 2.1. Clinical Characteristics and Laboratory Findings

Patients were mostly males (7/11) and the age at presentation ranged from 19 and 75 years, with a median age of 50.5 years. Five/eleven patients presented with typical constitutional B symptoms: weight loss (4/5), fever (3/5) and sweats (2/5). Asthenia, skin rash and stomatitis were reported at diagnosis in three, two and one patient, respectively. In one patient, the disease presented as skin rash, followed by fever 3 months later. In 3/11 cases, the disease presented with lymphadenopathy which was mostly diffuse; in 5/11, multiple lymphadenopathy occurred in association with splenomegaly; in 1/11 the disease presented with multiple lymphadenopathy, splenomegaly and hepatomegaly; in 1/11 cases, multiple lymphadenopathy developed 3 months after the initial presentation with skin rash; in 1/11, diffuse lymphadenopathy and splenomegaly appeared 2 months after an asymptomatic presentation. Thymus involvement was not observed in any patient. Blood test disclosed leukocytosis with eosinophilia at varying extent; the median white blood cell count (WBC:) and eosinophil count were 40,359/mm^3^ (range 18,000–72,830) and 7630/mm^3^ (1800–31,316), respectively. The median hemoglobin level was 12.7 g/dL (range 8.2–17.8) and median platelet count 175,272 (range 43,000–520,000). In 5/11 cases, the lactate dehydrogenase (LDH) level was elevated with a mean value of 610 U/L (range 386–897).

### 2.2. Bone Marrow Histological and Immunohistochemical Findings

In all cases, bone marrow (BM) biopsy revealed a hypercellular marrow with prevalence of myeloid cells in different stages of maturation and clear-cut increase in eosinophils. CD34-positive hematopoietic precursors were within normal limits (1–2%) in the majority of cases (10/11), with only one case showing increased CD34-positive cells (7%). In most cases (5/11), the erythroid lineage was reduced, with maturation defect and slight predominance of proerythroblasts and basophilic erythroblasts; in 1/11 (case n° 5), large aggregates of immature erythroid precursors along with immature myeloid precursors were noted and in 1/11, trilinear hyperplasia was present. Mild abnormalities were found in the megakaryocytic lineage, with either a mild increase in the number of megakaryocytes in 3/11 cases or a reduction in 1/11; nuclear lobulation defects of megakaryocytes were seen in 3/11 cases. In 4/11 cases, marrow fibrosis ranging from grade 1 to grade 3 was present. A minor B-lymphoblastic component of medium sized cells with high N/C ratio was identified in 2/11 cases, whereas in 1/11 cases, 5–10% of mast cells (tryptase+, CD117+, CD25−) were identified, mainly dispersed and occasionally in tiny paratrabecular aggregates, not meeting the current 2017 WHO criteria for a concomitant diagnosis of systemic mastocytosis (SM).

### 2.3. T-LBL Histological and Immunohistochemical Findings

**Figure 1:**
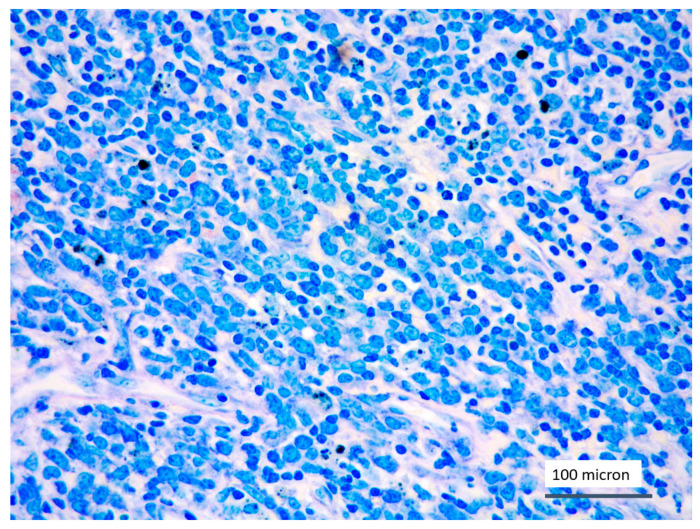
T-LBL medium-sized cells with high nuclear-cytoplasmic ratio and dispersed chromatin (case 5, Giemsa staining, magnification 400×).

**Figure 2:**
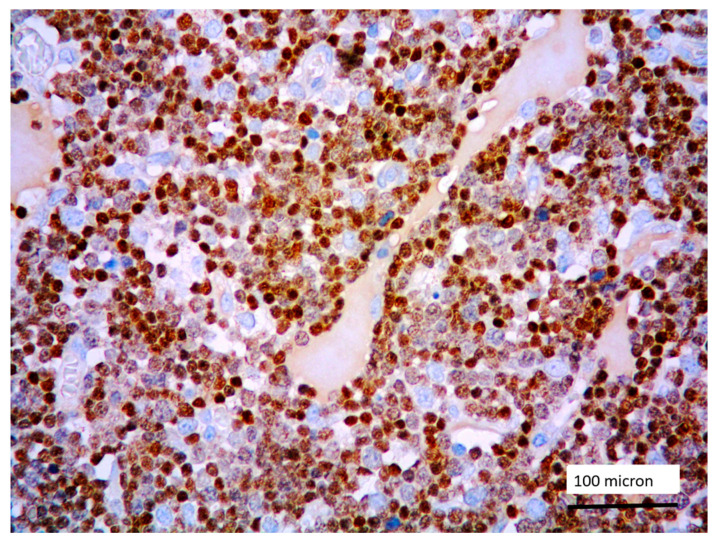
Lymphoblastic proliferation diffusely expressing TdT (case 5, magnification 400×).

**Figure 3:**
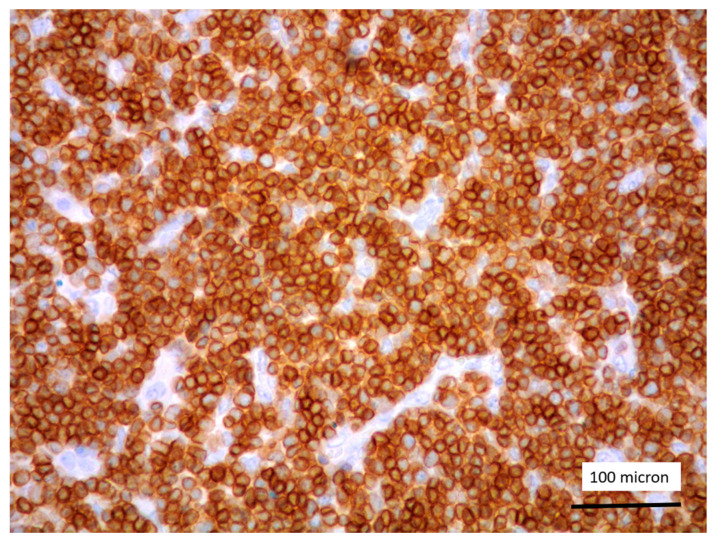
CD3 positivity of T-LBL (case 5, magnification 400×).

**Figure 4:**
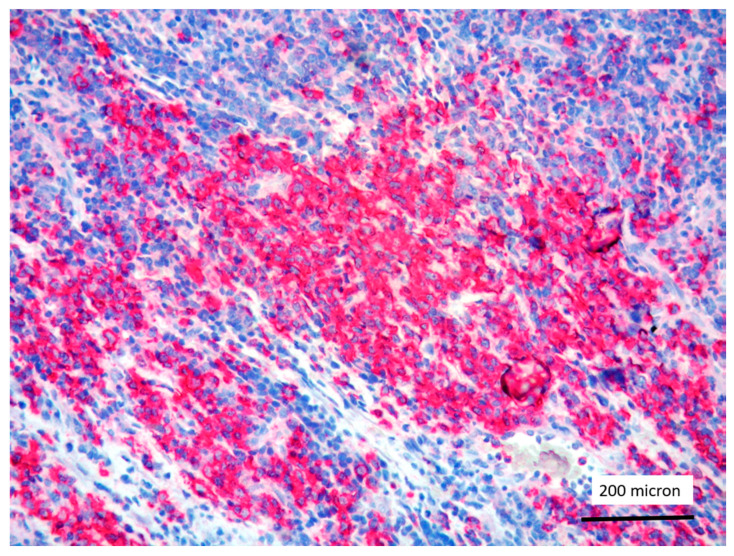
Immature myeloid cells, admixed to T-LBL, highlighted by myeloperoxidase (MPO) immunostaining (case 7, magnification 200×).

**Figure 5:**
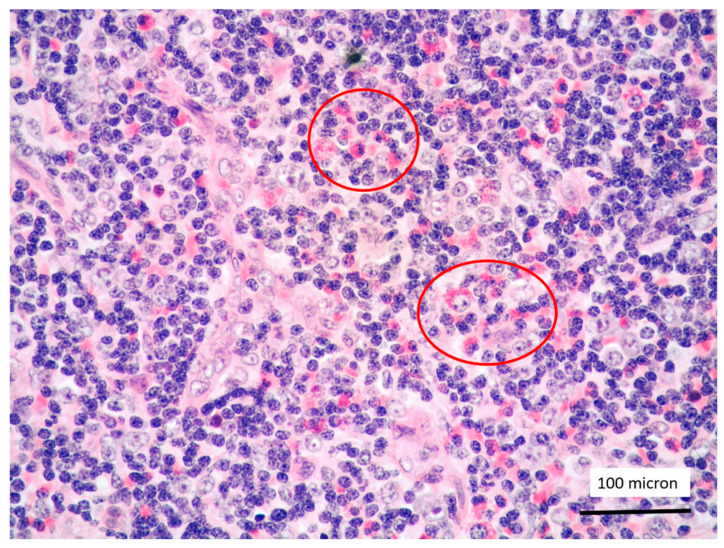
T-LBL containing a discrete component of eosinophils (within red circles) (case 7, Hematoxylin-Eosin staining, magnification 400×).

**Figure 6:**
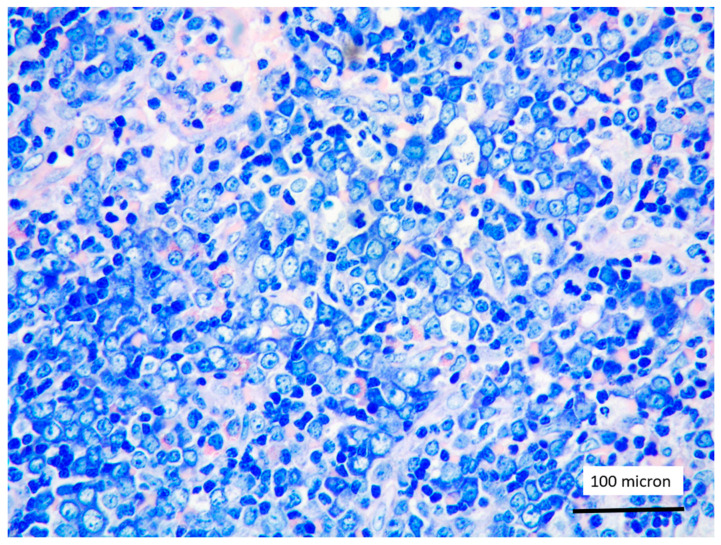
Aggregates of proerythroblasts in the context of T-LBL, in the PCM1-JAK2 rearranged case (case 5, Giemsa staining, magnification 400×).

**Figure 7:**
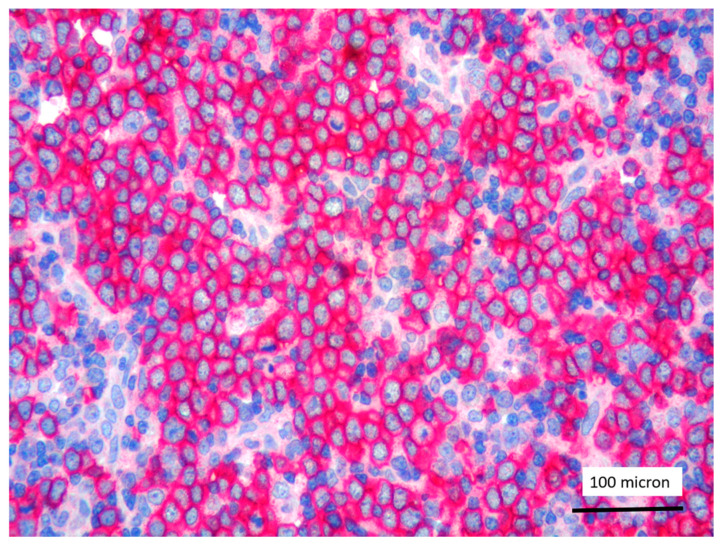
Glycophorin C highlighting proerythroblasts within T-LBL, in the PCM1-JAK2 rearranged case (case 5, immunostaining, magnification 400×).

**Figure 8:**
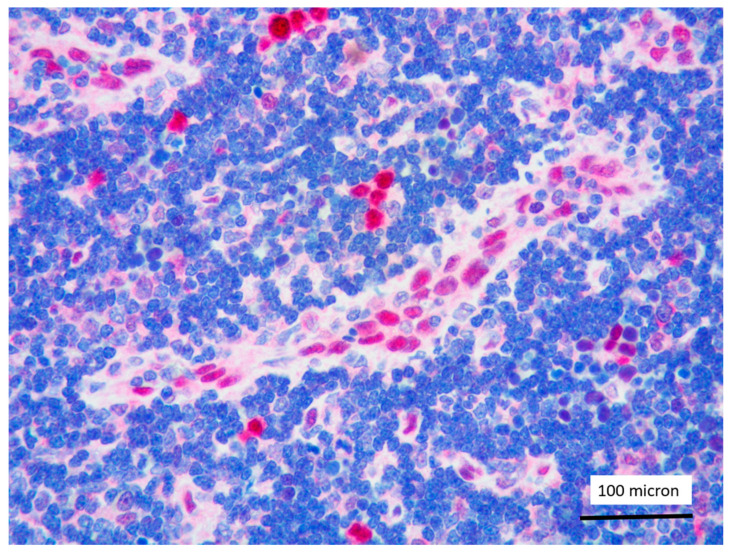
LMO2 negativity of T-LBL; endothelial cells as positive LMO2 controls (case 1, immunostaining, magnification 400×).

**Figure 9:**
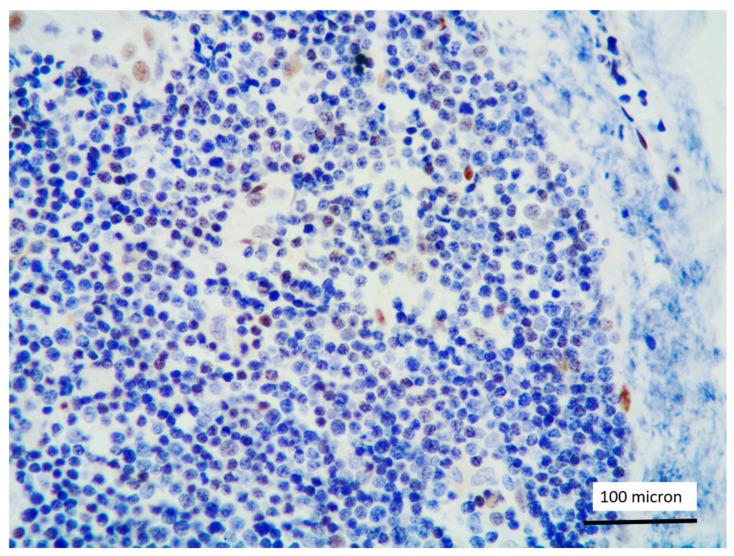
Partial and weak LMO2 expression of T-LBL (case 2, immunostaining, magnification 400×).

In all the cases, the lymph node histology and immunohistochemical profile were consistent with T-LBL diagnosis. The nodal architecture was effaced by a diffuse proliferation of medium-sized cells with dispersed chromatin and scarce cytoplasm (Figure 1) with high proliferative fraction and usually positive for TdT (Figure 2), CD1a and T-cell markers such as CD3 (Figure 3), CD2, CD8, CD5, CD7. B-cell markers (CD79α, CD20, PAX5 and CD22) were mostly negative; a weak CD79α co-expression was noted in 2/11 cases; myeloid (MPO, CD117, CD68KP1) and monocytic (CD68PGM1) markers were negative. In 5/11 cases, a minor component of immature myeloid cells was identified (Figure 4). Aggregates of mature eosinophils (Figure 5) admixed to the lymphoblastic proliferation were noted in 5/11 cases, whereas clusters of proerytroblasts (Figure 6 and Figure 7) were identified within T-LBL in 1/11 cases. Unexpectedly, LMO2 immunostaining was found to be negative in 9/11 T-LBLs (Figure 8); in the remaining 2 cases, LMO2 was partially expressed (less than 30% of cells) with either moderate or low degree of intensity, respectively (Figure 9).

### 2.4. Molecular Data

The karyotype and FISH analysis (see Supplementary Materials) identified FIP1L1-PDGFRA fusion gene in 2/11 cases, ZMYM2-FGFR1 fusion gene in 5/11, BCR-FGFR1 in 2/11, and PCM1/JAK2 in 1/11, whereas in the remaining 2 cases, any known genetic alteration was identified. Additionally, in case n° 7 (ZMYM2-FGFR1) gene fusion was confirmed by targeted NGS analysis. More detailed information concerning the cytogenetic abnormalities are reported in Supplementary Material.

### 2.5. Therapy and Outcome

Treatments administered to these patients were highly heterogeneous. The PDGFRA-related cases responded well to imatinib; in particular, the first case of our series is in complete molecular remission (CMR) at 10 years from diagnosis; the second PDGFRA-related case (n° 11) is quite recent and therapy with imatinib is still ongoing with good clinical and laboratory response. The majority of FGFR1-rearranged cases (n° 2, 4, 6, 9, 10) had a poor outcome, despite different intensive chemotherapy schemes. Details are provided in the Supplementary Materials. Case n° 7 represents the only FGFR1-related case with a good outcome, after 2 CVP (cyclophosphamide, vincristine, prednisone) cycles with no benefit, the FGFR-inhibitor pemigatinib was started and is still ongoing with good clinical and laboratory response. In two cases (n° 3 and n° 8) any known genetic alteration was identified; in case n° 3, the empiric treatment with imatinib (100 mg/daily), followed by the maintenance dose (100 mg/weekly) resulted in a complete hematological remission (CHR) at 10 years from diagnosis, despite the persistency of T-cell receptor gamma (TCR-γ) rearrangement by RT-PCR on peripheral blood; in case n° 8, in absence of a known genetic alteration, the chemotherapeutic approach with hyper-CVAD followed by allo-HSCT was used, obtaining a CHR at 5 years. Case n° 5 (PCM1-JAK2-positive) had a poor response to different chemotherapy schemes: 6-mercaptopurine (50 mg/m^2^/daily) plus low-dose cytarabine (40 mg/m^2^/daily), then FLAG (fludarabine, cytarabine, granulocyte colony-stimulating factor) with liposomal doxorubicin; due to refractory disease, the patient died before allo-HSCT.

## 3. Discussion

LMO2 belongs to a multigene family extremely conserved during evolution, containing two cystein-rich regions referred to as LIM domains; it encodes for the homonymous LMO2 protein playing a central role in angiogenesis and required for the development of normal haematopoiesis [9]. The highest expression of LMO2 was observed in normal GC lymphoid B cells, in GC-derived lymphoma and Burkitt lymphoma [10]. Additionally, LMO2 expression has been associated with a favorable prognosis in a subset of DLBCL with GC phenotype [11,12,13]. Conversely, LMO2 protein is not expressed during T-cell development; consequently, T-cell precursors of the thymus and T-cell areas of peripheral lymphoid organs are LMO2 negative [14]. The role of LMO2 as an oncogene capable of inducing T-cell lymphoblastic leukemia has been widely demonstrated in mouse models [17,18]; accordingly, several in vivo studies have suggested the role of LMO2 in the pathogenesis of T-LBL [19]. LMO2 seems to be a specific marker of transformed T-cell precursors compared with their normal counterparts [14]. In T-cell lymphoblastic lymphoma/leukemia, LMO2 overexpression is to a lesser extent attributable to the presence of t(11;14)(p13;q11) or t(7;11)(q35;p13) involving LMO2 gene or to cryptic deletions of negative regulators of its transcription [20,21]. To explain LMO2 overexpression in a large proportion of T-LBL, a more extensive involvement of LMO2 in T-cell precursors tumorigenesis by other still unknown mechanisms has been supposed [14].

T-LBL is an aggressive neoplasm of T-cell precursors, affecting mainly children and young adults and involving lymph nodes, BM and thymus [1]. The standard therapeutic approach for T-LBL involves multiagent chemotherapy regimens [22,23]. However, rare cases of T-LBL may develop in the context of M/LNs-Eo and rearrangement of tyrosine-kinase (TK) genes [1,3]. Accurate diagnosis and classification of T-LBL arising in this particular setting has important therapeutic implications.

The current 2017 WHO classification recognizes the following specific diseases: M/LNs-Eo with rearrangements of PDGFRA, PDGFRB and FGFR1 respectively and the provisional entity of myeloid/lymphoid neoplasms with PCM1-JAK2 rearrangement [1]. In addition to the aforementioned TK fusion genes, rearrangements involving FLT3, ABL1 and LYN genes have also been reported in M/LNs-Eo, although not formally included in the current WHO classification [24,25,26]. The typical presentation of M/LNs-Eo consists of a MPN with associated variable degree of eosinophilia, but clinical manifestations are proteiform [27], involving simultaneously or sequentially the myeloid and or/lymphoid lineages, even in individual patients [1,2,3,4]. Each subtype has a preferential disease presentation and eosinophilia or hypereosinophilia (absolute eosinophilic count of 1.5 × 10^9^ L or more) is common, but not invariably present. PDGFRA-rearranged cases often present as chronic eosinophilic leukemia (CEL) and, less commonly, as AML or T-LBL [1,3,28].

PDGFRB-rearranged cases preferentially manifest as chronic myelomonocytic leukemia (CMML), and less frequently as CEL, MPN or atypical chronic myeloid leukemia (aCML) [1,3,29,30]. In the PDGFRB category, LBL occurrence is rare [1,15]. FGFR1-related cases most often present as T-LBL and less commonly as CEL, B-LBL and AML [1,3,31,32,33]. PCM1-JAK2-related cases can manifest as CEL, aCML, primary myelofibrosis and rarely as AML or B and T-LBL [34].

In the present series, we analyzed the clinic-pathological features of 11 cases of M/LNs-Eo (2 FIP1L1-PDGFRA-rearranged; 5 ZMYM2-FGFR1-rearranged; 1 BCR-FGFR1-rearranged; 1 PCM1/JAK2-related; and 2 without any known genetic alterations). In all the cases, the disease presented as T-LBL associated with MPN and eosinophilia. We observed a different LMO2 expression in T-LBL arising in the setting of M/LNs-Eo compared to sporadic T-LBL.

It is crucial to identify T-LBL occurring in M/LNs-Eo, given the exquisite responsiveness to different TKIs of some of these disorders. Besides some helpful morphological clues (i.e., eosinophils and/or immature myeloid elements in the context of T-LBL [3,35], proerythroblasts admixed to T-LBL, as well as the classical BM “triad” including hypercellularity with eosinophilia, clusters of erythroid precursors and fibrosis in PCM1-JAK2-rearranged cases [3,36]), the immunohistochemical search for LMO2 may represent an aid to identify T-LBL occurring in the setting of M/LNs-Eo.

T-LBL almost universally overexpress LMO2 [14,15]. Unexpectedly, in our series, the majority of T-LBL occurring in the context of M/LNs-Eo was LMO2 negative, with only 2 cases showing partial LMO2 expression with moderate and low degree of intensity, respectively. Jevremovic et al. reported that LMO2 represents a marker commonly expressed by T-LBL and absent in thymocytes of normal thymus and thymomas [15]. Since there is no specific immunophenotypic profile to distinguish thymocytes or thymic epithelial tumors from neoplastic T lymphoblasts, LMO2 is currently considered a useful marker in discriminating thymoma from T-LBL, being almost universally expressed in T-LBL [15]. Recently Brar et al. identified LMO2 as a sensitive and specific marker for differentiating T-LBL from iT-LBP, which were found to be LMO2-negative [16]. The absence of LMO2 expression in iT-LBP was considered by Brar et al. consistent with a thymic origin for iT-LBP as T-lymphoblasts in the thymus do not express LMO2 [16].

In our study, we would suggest that LMO2 immunostaining, as part of the diagnostic panel for T-LBL, may represent a potentially useful marker to identify T-LBL developing in the context of M/LNs-Eo. In this particular setting, T-LBL shows an immunohistochemical profile overlapping with cortical thymocytes and iT-LBP, possibly reflecting different molecular patterns involved in the pathogenesis of T-LBL arising in the setting of M/LNs-Eo.

## 4. Materials and Methods

We retrospectively collected clinical, histopathological and molecular data of 11 cases of T-LBL, occurring in the setting of M/LNs-Eo, diagnosed at six of our institutions between 2006 and 2020. All data were critically re-evaluated, according to the latest 2017 WHO criteria [1]. Cytogenetic and molecular analyses were performed at respective institutions at the time of diagnosis as part of the routine clinical work-up. 4-μm-thick sections were cut from formalin-fixed and paraffin-embedded blocks of lymph nodes with T-LBL. Immunohistochemical tests included the determination of LMO2. In 10/11 cases, the anti-LMO2 antibody clone SP51 (Spring Bioscience, Pleasanton, CA, USA) was applied at a dilution 1:400 for 30 min, following antigen retrieval in a PT link at 92 °C (high pH solution) for 5 min. The detection system used was the ultra-view CC1 on a Ventana platform (Ventana Medical Systems, Oro Valley, AZ, USA) in 8/11 cases and the Dako REAL detection system Alkaline Phosphatase/RED on a Dako AutoStainer Plus (Agilent Dako, Santa Clara, CA, USA) in the remaining two. In one further case, the LMO2 antibody used was LMO2, clone 1A9-1 (Roche, Basel, Switzerland), undiluted, CC1 37 °C (high pH) 32 min + 30 min of primary antibody incubation, with detection system Opti-VIEW Universal DAB detection Kit, platform Bench Mark Ultra (Ventana Medical Systems, Inc., Oro Valley, AZ, USA).

To reduce bias in data analysis, immunohistochemical results were blindly re-evaluated at two institutions (Pathology Unit, Azienda Ospedaliera Santa Maria di Terni, University of Perugia, Terni, Italy and Haematopathology Unit, IRCCS Azienda Ospedaliero-Universitaria di Bologna, Bologna, Italy). No discrepancies were recorded between the estimates at the two Institutions.

## 5. Conclusions

To the best of our knowledge, this is the first study investigating LMO2 expression by immunohistochemistry in T-LBL cases arising in the context of M/LNs-Eo. On the basis of our findings, immunohistochemical LMO2 expression, along with some morphological clues, may help to identify cases of T-LBL in the setting of M/LNs-Eo, stating that this group of disorders may be easily underdiagnosed due to both its rarity and proteiform clinical presentation. In conclusion, our study may be considered an exploratory analysis for more extensive studies including further cases and more in-depth molecular analysis to better address the frequent negative LMO2 immunohistochemical expression in T-LBL developing in the context of M/LNs-Eo.

## Figures and Tables

**Table 1:** Clinic-pathological features of T-LBL cases in M/LNs-Eo.

Case: Age/Sex	Clinical Presentation	Peripheral Blood	BM Histology/ Lymph Node Histology	LMO2 Expression	Molecular Analyses (on BM)	Fusion Gene	Therapy Outcome
Case 1: 75/M	Dyspnea, asthenia, weight loss, skin rash, splenomegaly, lymphadenopathy (axillary, inguinal)	WBC: 20,200 Eo: 12,400 PTL: 138,000 Hb: 8.2 MCV: 109	CEL. Grade 3 fibrosis. T-LBL with minor immature myeloid component	Negative	46XY del(4)(q12;q12) *BCR/ABL1* negative JAK2V617F negative Chromosome 16 inversion negative	*FIP1L1-PDGFRA*	Imatinib (100 mg/die × 2 days; then 200 mg/die); maintenance therapy with CMR at 10 yrs
Case 2: 66/F	Splenomegaly, diffuse lymphadenopathy	WBC: 25,600 N: 19,200 M: 1024 Eo: 3072 RBC: 6,820,000 Hb: 15.9 HCT: 49.5 PTL 387,000 LDH: 527	MPN with eosinophilia. Grade 1 fibrosis. T-LBL	Weak and partial	46XY t(8;13)(p11;q12) *BCR/ABL1* negative JAK2/V617F negative *FI1L1-PDGFRA* negative	*ZMYM2-FGFR1*	Hyper-CVAD Exitus at 2 mo
Case 3: 40/M	Skin rash. 3 mos later: skin papules, fever; lymphadenopathy (cervical, inguinal)	WBC: 28,880 N: 59% L: 21 M: 4% Eo: 7% My 4%: Meta 5% Hb: 10.8 PTL 180,000 3 mos later: WBC: 42,420 N: 31,391 Eo: 6363 Hb: 10.5	Reactive myeloid hyperplasia in d.d. with CML T-LBL with Eo	Negative	*TCRγ* + *BCR/ABL1* negative JAK2V617F negative MPL negative *PDGFRA* negative *TET2/4q24* negative *PDGFRB/5q33* negative *FGFR1/8p21* negative JAK2/9p24 negative ETV6/12p13 negative	Unknown genetic alteration	Imatinib (100 mg/die); maintenance therapy (100 mg/weekly) CHR at 10 years
Case 4: 56/M	Diffuse lymphadenopathy	WBC: 57,000 N: 33,000 L: 5800 M: 8900 Eo: 8600 PTL: 178,000 Hb: 13.3 LDH 897	MPN with eosinophilia + B-LBL component. T-LBL with Eo	Negative	46XY t(8;13)(q24;q12); del(9)(q22) der(5) BCR/ABL1 negative JAK2V617F negative *PDGFRA* negative MYC(8q24) negative TCRγ negative	*ZMYM2-FGFR1*	No therapy. Exitus shortly after diagnosis
Case 5: 19/M	Lymphadenopathy (cervical, sub-mandibular), splenomegaly. Then diffuse lymphadenopathy	WBC: 61,440 N: 46,990 M: 3330 L: 6850 Eo: 3330 B: 1610 Hb: 12.9 PTL: 85,000 LDH: 658	MPN with eosinophilia+ erythroid precursors + fibrosis T-LBL with Eo and proerythroblasts	Negative	t(8;12) BCR/ABL1 negative PDGFRA negative PDGFRB negative FGFR1 negative	*PCM1/JAK2*	6-mercapto (50/mg/m^2^/die) + Cyta. (40/mg/m^2^/die): hydroxyurea + prednisone; FLAG + Myocet. Exitus before allo-HSCT
Case 6: 19/M	Stomatitis, fever, diffuse lymphadenopathy splenomegaly, hepatomegaly	WBC: 18,000 with neutrophilia eosinophilia (3600) left shifting PTL: 80,000 Hb: 12	MPN with eosinophilia T-LBL with Eo and minor immature myeloid component	Negative	t(8;13)(p11;q12) t(14;21)(q22;q22) t(8;13) IGH + IGL + TCRγ+	*ZMYM2-FGFR1*	GRAAL-LYSA LL03 + allo-HSCT CMR, then AML with exitus despite salvage CT
Case 7: 74/F	Fever, night sweats, weight loss, lymphadenopathy (cervical)	WBC: 42,000 N: 28,700 Eo: 5300 B: 600 PTL: 50.00 Hb: 13 LDH: 386	MPN with eosinophilia + B-LBL component + mast cells (5–10%). Grade 2 fibrosis T-LBL with Eo and minor immature myeloid component	Negative	t(8;13)(p11;q12) *BCR/ABL1* negative *PDGFRA* negative *PDGFRB* negative KIT D816V negative	*ZMYM2-FGFR1*	CVP (2 cycles) with no benefit. Pemigatinib (ongoing) with benefit.
Case 8: 49/M	Asthenia, sweats, weight loss, splenomegaly, diffuse lymphadenopathy	WBC: 47,000 N: 40% prom: 4% My: 8% Meta: 8% Eo: 3.20 Hb: 13.8 PTL: 520,000	MPN with eosinophilia T-LBL + minor immature myeloid component	Negative	Normal 46XY karyotype *BCR/ABL1* negative *PDGFRA* NP *PDGFRB* NP *FGFR1* NP	Not detected	Hyper-CVAD + allo-HSCT. cGVHD (steroid, rituximab) CHR at 5 yrs. Mycophenolate mofetil + extracorporeal photopheresis (for cGVHD)
Case 9: 49/F	Lymphadenopathy (cervical)	Hb: 17.8 HCT: 54% WBC: 31,000 Eo: 30% PTL: 217,000 LDH: 586	MPN with eosinophilia T-LBL	Negative	t(8;13)(p11;q12)	*ZMYM2-FGFR1*	Hyper-CVAD, then Cyta (3 g/m^2^) followed by busulfan plus cycloph and autologous HSCT; then anti-CD52 therapy. Disease progression with exitus in 2 mos
Case 10: 51/F	Asymptomatic at presentation. Hydroxyurea with no benefit; 2 mos later: diffuse lymphadenopathy, splenomegaly	WBC: 40,000 Eo: 1800 PTL: 43,000 Hb: 12 FC: aberrant T-cell population (7.89%): CD7+ sCD3− CD4−/+ CD8+ CD16− CD56+ CD5+ CD2+ cyCD3+	MPN/MDS with 7% CD34+ T-LBL + minor immature myeloid component	Moderate/ partial	4 clones: t(8;22)(p11;q11); t(8;22)(p11;q11)+ trisomy 19; der(22)+ t(8;22)(p11;q11)+ trisomy 19 normal XX clone *BCR/ABL1* negative MPL negative JAK2V617F negative	*BCR-FGFR1*	Hydroxyurea. Due to lymphadenopathy (T-LBL) and splenomegaly Hyper-CVAD with transient response; FLA with no response and exitus
Case 11: 58/M	Asthenia, fever, weight loss, lymphadenopathy, splenomegaly	WBC: 72.8 N: 34% L: 5% Eo: 43% Hb: 10.6 PTL: 50,000; then progressive anemia (Hb: 9) and Eo: 30,000	MPN with eosinophilia T-LBL	Negative	*FIP1L1-PDGFRA* positive *CR/ABL1* negative JAK2V617F negative Calreticulin negative MPL negative	*FIP1L1-PDGFRA*	Imatinib (100 mg) still ongoing with Eo. count decrease (170) and lymphadenopathy reduction

allo-HSCT: allogenic hematopoietic stem cell transplantation; AML: acute myeloid leukemia; B-LBL: B-lymphoblastic lymphoma; BM: bone marrow; CEL: chronic eosinophilic leukemia; CHR: complete hematological remission; CML: chronic myeloid leukemia; CMR: complete molecular remission; cGVHD: chronic graft versus host disease; Cycloph: cyclophosphamide; Cyta: Cytarabine; CT: chemotherapy; DD: differential diagnosis; EBER: Epstein Barr virus encoded RNA; Eo: eosinophils; FC: flow cytometry; FLA: fludarabine plus cytarabine; FLAG: fludarabine, cytarabine, granulocyte-colony stimulating factor; GRAAL-LYSA LM03: vincristine, daunorubicin, cyclophosphamide, L-asparaginase, methotrexate, cytarabine, depo-medrol; Hb: hemoglobin; HCT: hematocrit; Hyper-CVAD: cyclophoshamide, dexamethasone, methotrexate, doxorubicin, vincristine, cytarabine; HSCT: hematopoietic stem cell transplantation; IGH: immunoglobulin heavy chain; IGL: immunoglobulin light chain; L: lymphocytes; 6-mercapto: 6-mercaptopurine; MCV: mean corpuscular volume; MDS/MPN: myelodysplastic/myeloproliferative neoplasms; Meta: metamyelocytes; mod: moderate; M/LNs-Eo: myeloid/lymphoid neoplasms with eosinophilia; M: monocytes; My: myelocytes; Myocet: nonpegylated liposomal doxorubin; Mo: month; Mos: months; MPN: myeloproliferative neoplasm; N: neutrophils; Neg: negative; NP: not performed; prom: promyelocytes; Pos: positive; PTL: platelets; RBC: red blood cell; TCR: T-cell receptor; T-LBL: T-lymphoblastic lymphoma; WBC: white blood cells; yrs: years.

## Data Availability

The data presented in this study are available on request from the corresponding author.

## Supplementary Materials

The following are available online at https://www.mdpi.com/article/10.3390/cancers13123102/s1, Text S1: Presentation of the eleven clinical cases.
