# Supplementary material for: T-Cell Lymphoblastic Lymphoma Arising in the Setting of Myeloid/Lymphoid Neoplasms with Eosinophilia: LMO2 Immunohistochemistry as a Potentially Useful Diagnostic Marker
Source: Cancers (Basel). 2021 Jun 21;13(12):3102. doi: 10.3390/cancers13123102 (PMC8234657; doi:10.3390/cancers13123102)
Supplement: Supplementary file 1 [file cancers-13-03102-s001.zip › cancers-1185825-supplementary.pdf]

cancers MDPI

Supplementary Materials: T-Cell Lymphoblastic Lymphoma Arising in the Setting of Myeloid/Lymphoid Neoplasms with Eosinophilia: LMO2 Immunohistochemistry as a Potentially Useful Diagnostic Marker

Magda Zanelli, Giuseppe G. Loscocco, Elena Sabattini, Maurizio Zizzo, Francesca Sanguedolce, Luigi Panico, Daniela Fanni, Raffaella Santi, Cecilia Caprera, Cristiana Rossi, Alessandra Soriano, Alberto Cavazza, Alessandro Giunta, Cristina Mecucci, Alessandro M. Vannucchi, Stefano A. Pileri and Stefano Ascani

Text S1. Presentation of the eleven clinical cases

Case 1

A 75-year-old male presented with dyspnea on exertion, asthenia, unintentional weight loss of 30 kg in 12 months and erythematous skin rash on arms and face. Night sweats, fever and itching were absent. Computerized tomography (CT) scan disclosed splenomegaly, axillary and inguinal lymphadenopathy. Peripheral blood (PB) tests revealed leukocytosis (20,200/mm^3), severe eosinophilia (12,400/mm^3), mild thrombocytopenia (138,000/mm^3) and mild macrocytic anemia (Hb: 8.2 g/dL; MCV: 109 fl). Clinical work-up for infectious, allergic and autoimmune conditions was negative. Histologically, the architecture of the excised axillary lymph node was effaced by a diffuse proliferation of medium-sized cells with dispersed chromatin and scarce cytoplasm, TdT+, CD1a+, CD3+, CD2+, CD8+, CD5+, CD7+ CD79α+ (weak) and CD34−, LMO2−, CD4−, CD20−, PAX5−, CD22−, perforin−, MPO−, CD117−, CD68/KP1−, CD68/PGM1−, with high proliferation index (Ki67: 98%). Less than 5% of MPO+, CD68/KP1+ and CD117+ blasts were identified. The diagnosis of T-LBL with a small immature myeloid component was made. Bone marrow (BM) was hypercellular (90% cellularity) with prominent myeloid hyperplasia, moderate excess of myelocytes and promyelocytes and increased eosinophils (35%). No increase of CD34-positive hematopoietic precursors was present. The erythroid lineage was reduced, with prevalence of proerythroblasts and basophilic erythroblasts; few micro-megakaryocytes were present. The lymphoid infiltrate as well as mast cells were within normal limits. Grade 3 fibrosis was present. Conventional cytogenetic analysis revealed a 46, XY karyotype. Reverse transcriptase-polymerase chain reaction (RT-PCR) detected del(4)(q12;q12), resulting in *FIP1L1-PDGFRA* fusion gene. *BCR/ABL1* and JAK2 V617F mutations were negative. Chromosome 16 inversion was not identified by FISH analysis. The deletion in region 12 of chromosome 4 long arm (del 4q12) containing the cysteine-rich hydrophobic domain 2 (CHIC2), which is a surrogate marker for the *FIP1L1-PDGFRA* fusion gene, was detected by FISH.

The final diagnosis was MPN consistent with CEL with *FIP1L1-PDGFRA* fusion gene, associated with T-LBL. The patient was started on imatinib (100 mg/daily for 2 days and then 200 mg/daily), resulting in complete molecular remission (CMR) after one year. The patient continues the maintenance therapy and remains in CMR at 10 years from diagnosis.

Case 2

A 66-year-old asymptomatic woman was incidentally discovered on blood tests to have increased erythrocytes (6,820,000/mm^3), with hematocrit (HCT), Hb and platelets (PTL) at upper levels (HCT: 49.5%; Hb: 15.9 g/dL; PTL: 387,000/mm^3). Leukocytosis (25,600/mm^3), with neutrophilia (19,200/mm^3), monocytosis (1,024/mm^3) and moderate eosinophilia (3,072/mm^3) was disclosed. LDH was elevated (527 U/L). Diffuse

supradiaphragmatic and subdiaphragmatic lymphadenopathy and splenomegaly were identified by CT scan. Lymph node biopsy showed a diffuse involvement by T-LBL with the following immunophenotype: TdT+, CD1a+, CD99+, CD3+, CD4+, CD5+, CD8+/−, LMO2+/− (mild and partial expression), CD34−, CD10−, CD56−, CD79a−, CD20−, Bcl6− and high proliferative index (Ki67: 80–90%). BM biopsy revealed an hypercellular marrow with increase of myeloid cells in different stages of maturation and numerous eosinophils. CD34-positive hematopoietic precursors were normal (1–2%). The erythroid lineage was reduced with slight excess of proerythroblasts and basophilic erythroblasts. A slight increase of normal, non-clustering megakaryocytes was noted. Monocytes, mast cells and lymphocytes were normal. Grade 1 fibrosis was present. *BCR/ABL1* fusion gene, V617F JAK2 mutation and *FIP1L1-PDGFRA* were absent. Cytogenetic analysis revealed a 46, XY karyotype with t(8;13)(p11;q12), compatible with the presence of *ZMYM2-FGFR1* fusion gene.

BM histology along with t(8;13)(p11;q12) was in keeping with M/LNs-Eo with *FGFR1* rearrangement in blast phase as T-LBL. The patient received hyper-CVAD regimen (cyclophosphamide, dexamethasone, methotrexate, doxorubicin, vincristine, cytarabine), with no clinical improvement and died 2 months later.

Case 3

A 40-year-old male presented with few months history of pruritic skin rash on face and upper extremities treated with steroid therapy without benefit. Blood tests showed leukocytosis (28,880/mm^3), mild anemia (Hb 10.8 g/dL) and mild thrombocytopenia (180,000/mm^3). The white blood cell (WBC) differential showed 59% neutrophils, 21% lymphoid cells, 4% monocytes, 0% basophils, 7% eosinophils, 4% myelocytes, 5% metamyelocytes. BM aspirate revealed striking myeloid lineage expansion with left shifting and 4% of precursors; the other hematopoietic elements were normal. BM was hypercellular (90%) with pronounced granulocytic hyperplasia and eosinophilia, in absence of abnormal features of the erythroid and megakaryocytic lineages. Blasts were not increased. BM histology could be in keeping with either reactive myeloid hyperplasia or chronic myeloid leukemia (CML). Translocation t(9;22)(q34;q11) and *BCR-ABL1* fusion gene along with JAK2 V617F and MPL mutations were absent. Clonal T-cell receptor γ (TCR-γ) gene rearrangement was identified by RT-PCR. FISH analyses for *PDGFRA*/4q12, *TET2*/4q24, *PDGFRB*/5q33, *FGFR1*/8p21, JAK2/9p24, ETV6/12p13 were all negative. Three months later, the patient developed multiple erythematous skin papules and pustules on trunks and legs poorly responsive to topic antibiotics. Blood test showed slight anemia (Hb: 10.5 g/dL), leukocytosis (42,420/mm^3), neutrophilia (31,391/mm^3) and severe eosinophilia (6,363/mm^3). Due to the appearance of inguinal and cervical lymphadenopathy and serotinous fever (37.5 °C), an excisional lymph node biopsy was performed. The lymph node architecture was totally effaced by small to medium-sized cells TdT+, CD1a+, CD3+, CD4+, CD8+, CD10−, MPO−, CD68PGM1−, CD34−, CD20−, LMO2− with high index of proliferation (Ki67 80%). Aggregates of mature eosinophils were admixed to the lymphoblastic proliferation. The histology was consistent with T-LBL, with associated eosinophilia. Despite the absence of investigated *PDGFRA*, *PDGFRB*, *FGFR1*, JAK2 and ETV6 rearrangements, the clinical and laboratory findings along with BM and lymph node histology were consistent with M/LNs-Eo and associated T-LBL. A complete hematological remission (CHR) with the empiric use of imatinib (100 mg/daily) was obtained. The patient is continuing imatinib at the maintenance dose (100 mg/weekly) and remains in CHR at 10 years from diagnosis, despite the persistence of TCR-γ rearrangement by RT-PCR on PB.

Case 4

A 56-year-old male presented with diffuse lymphadenopathy. PB examination showed leukocytosis (leukocytes: 57,000/mm^3; neutrophils: 33,000/mm^3 (58%); lymphocytes: 5,800/mm^3 (10%); monocytes: 8,900/mm^3 (15%); eosinophils: 8,600/mm^3

(15%)). PTL count (178,000/mm^3) and Hb (13.3 g/dL) were within normal limits. LDH was increased (897 U/L). The inguinal lymph node histology showed a diffuse effacement by T-LBL with the following phenotype: TdT+, CD1a+, CD3+, CD4+, CD8 rare cells+, LMO2−, PAX5−, CD79α−, CD20−. Scattered eosinophils were present. No TCRγ was identified. BM biopsy showed: high cellularity (95%); granulocytic expansion with preserved maturation and clear-cut increase in eosinophils; reduced erythropoiesis and megakaryopoiesis; dysmorphic megakaryocytes. There was admixed a minor lymphoblastic population of medium sized cells with high N/C ratio (TdT+, Pax5+, CD79a+, CD10+, CD34−, CD117−, CD3−, MPO−). The diagnosis was: coexistence of MPN with eosinophilia and B-LBL. Chromosome binding analysis on bone marrow cells identified a 46 XY karyotype; t(8;13)(q24;q12); del(9)(q22), der(5). *BCR/ABL1* and JAK2V617F were negative. FISH analysis disclosed *FGFR1* rearrangement (8p11) as follows: *FGFR1* rearrangement in 45% of interphase nuclei; on metaphases: red telomeric probe on der(13), centromeric green probe retained on p11 region of der(8). MYC (8q24) rearrangement was negative as well as *PDGFRA-FIP1L1*. A possible novel three way variant translocation of t(8;13)(p11;q12) involving 8q24 region on der(8) occurred. The complex rearrangement masked the 8p11 involvement by CBA and only FISH analysis ascertained *FGFR1* rearrangement. The aase was compatible with the presence of *ZMYM2-FGFR1* fusion gene. The patient's clinical conditions rapidly deteriorated and he died shortly after.

Case 5

A 19-year-old male presented with occipital and sub-mandibular lymphadenopathy and splenomegaly. Blood tests revealed leukocytosis (61,440/mm^3); neutrophils: 46,990/mm^3; monocytes: 3330/mm^3; lymphocytes: 6,580/mm^3; eosinophils: 3,330/mm^3; basophils: 1,610/mm^3; mild anemia (Hb 12.9 g/dL) and low PTL count (85,000/mm^3). LDH was elevated (658 U/L). PB smear identified numerous myeloid cells, including a discrete number of myelocytes and metamyelocytes. BM aspirate was hypercellular with prevalence of myeloid cells in different stages of maturation (81%) and numerous eosinophils; erythroid cells (15%); lymphocytes (4%). BM was hypercellular with an eosinophilic infiltrate, large aggregates of immature precursors of both erythroid (glycophorin A+) and myeloid (MPO+, CD34−, CD117−) lineages and fibrosis.

The features were suggestive of MPN. *BCR/ABL1* rearrangement was negative. Due to marked increase of leukocyte count (123,000/mm^3, neutrophils 98,500/mm^3, lymphocytes 9,700/mm^3, monocytes 7,090/mm^3, eosinophils 5,520/mm^3, basophils: 2,110/mm^3) and appearance of diffuse lymphadenopathy, cytoreductive therapy with 6-mercaptopurine (50 mg/m^2/daily) and cytarabine (40 mg/m^2/daily) was started. The inguinal lymph node histology revealed a T-LBL: CD3+, CD2+/−CD5+/−, CD7−/+, TdT+, CD79*α*−, CD1a+, CD4+, CD8+, CD34−, LMO2− associated with aggregates of both eosinophils and proerytroblasts. The case was highly suspicious for M/LNs-Eo. *PDGFRA*, *PDGFRB*, *FGFR1* rearrangements were negative. 6-mercaptopurine was stopped and hydroxyurea plus prednisone started. Subsequently, t(8;12) was identified and FISH analysis revealed *PCM1-JAK2* rearrangement. Due to worsening of clinical conditions, the patient started the combination chemotherapy consisting of fludarabine, cytarabine and granulocyte colony-stimulating factor (FLAG) with nonpegylated liposomal doxorubicin (Myocet). Unfortunately, the clinical conditions rapidly deteriorated and the patient expired before allogenic hematopoietic stem cell transplantation (allo-HSCT).

Case 6

A 19-year-old male with a recent history of stomatitis and recurrent fever (39 °C) poorly responsive to antibiotics, presented with multiple cervical, thoracic and abdominal lymphadenopathy, mild splenomegaly (15 × 8 cm) and hepatomegaly. PB tests revealed moderate leukocytosis (18,000/mm^3) with neutrophilia and left shifting with myelocytes and metamyelocytes. Moderate eosinophilia (3,600/mm^3) was present, without peripheral blasts. PTL count was low (80,000/mm^3) and hemoglobin below normal value (Hb

12g/dL). The excised lymph node was totally effaced by a proliferation of medium-sized cells lacking nucleoli TdT+, CD99+, CD3+, CD2+, CD5+, CD7+/− (weak), CD4+, CD8+/−, LMO2−, CD79α + (weak), CD20−, PAX5−, BCL6−, CD10−, CD34−, CD117−, CD56−, CD57−, CD21−, CD23−, CD30−, MUM1/IRF4−, LMO2−. In situ hybridization for Epstein Barr-virus encoded RNA (EBER) was negative. Clusters of eosinophils and occasional MPO+ immature myeloid elements were admixed. The diagnosis of T-LBL was made. BM biopsy showed hypercellularity (90–95%) with an expanded and normally maturing myeloid lineage and an associated moderate eosinophilia. CD34+ precursors were normal (1–2%). The erythroid lineage was reduced, with maturation defect and slight predominance of proerythroblasts and erythroblasts. Megakaryopoiesis was slightly increased. Clusters of CD303+, CD123+ plasmacytoid dendritic cells were present. Molecular analyses on marrow aspirate identified TCR-γ rearrangement along with Immunoglobulin heavy chain (IGH) and Immunoglobulin light chain κ (IGLκ) rearrangements. JAK2V617F and MPL mutations were absent. Cytogenetic analysis revealed in 18/20 metaphases a complex karyotype with a translocation involving chromosome 8 short arm and chromosome 13 long arm, t(8;13)(p11;q12), and a translocation involving chromosome 14 long arm and chromosome 21 long arm, t(14;21)(q22;q22). In 3/18 metaphases, 48 chromosomes with t(8;13) duplication were identified. The final diagnosis was M/LNs-Eo and T-LBL associated with the presence of *ZMYM2-FGFR1* fusion gene. The patient received induction chemotherapy according GRAAL-LYSA LL03 (vincristine, daunorubicin, cyclophosphamide, L-asparaginase, methotrexate, cytarabine, depo-medrol) scheme, followed by allo-HSCT. After obtaining CMR, the patient developed AML with t(8;13) plus adjunctive, complex chromosomal abnormalities. Despite intensive salvage chemotherapy for AML, the patient rapidly progressed and died.

Case 7

A 74-year-old female was referred for fever (37.5 °C), night sweats, unintentional weight loss and painful cervical lymphadenopathy non responsive to antibiotics and steroids. PB exams showed leukocytosis (42,000/mm^3) with neutrophilia (28,700/mm^3), eosinophilia (5,300/mm^3) and basophilia (600/mm^3), together with low PTL count (50,000/mm^3) and normal hemoglobin level (Hb 13 g/dL). LDH was moderately elevated (386 U/L). CT scan revealed diffuse lymphadenopathy and splenomegaly. The surgically removed cervical lymph node showed architecture effacement by a diffuse proliferation extending to the perinodal adipose tissue, made up of medium-sized lymphoid cells with high N/C ratio and dispersed chromatin TdT+, CD99+, CD1a+/−, CD10−/+, CD34−, CD2+, CD3+, CD5+, CD7+, CD4+/−, CD8 (partial, weak expression), BCL6+/−, CD30 (occasional, weak expression), CD79α−, CD117−, LMO2−, c-MYC−, with index of proliferation (Ki67 95%). Few immature myeloid cells (MPO+, CD117+) and numerous eosinophils were present. The features were consistent with T-LBL. BM was hypercellular (90%) with predominant granulopoiesis and hypereosinophilia; markedly reduced erythropoiesis with excess of proerythroblasts and basophilic erythroblasts; slightly increased megakaryocytes with nuclear maturation defects. A small neoplastic clone of B lymphoblasts (TdT+, CD34+, PAX5+, CD79α+, CD10+, CD20−, CD3−, CD1a−, MPO−) representing 5–10% of cellularity was present. 5–10% of mast cells (tryptase+, CD117+, CD25−) either dispersed or in tiny paratrabecular aggregates were also identified; grade 2 fibrosis in the areas of mast cells clusters was described. *BCR/ABL1*, *PDGFRA* and *PDGFRB* rearrangements were negative. Cytogenetic studies revealed in 16/21 metaphases the presence of a translocation involving chromosome 8 short arm and chromosome 13 long arm, t(8;13)(p11;q12). FISH analysis identified the rearrangement involving *FGFR1* gene in 100% of cells examined. Next-generation sequencing (NGS) analysis confirmed the presence of *ZMYM2-FGFR1* gene rearrangement on both PB and BM aspirate. Considering the BM histology, nodal T-LBL and the molecular findings, a diagnosis of M/LNs-Eo with *FGFR1* rearrangement was made. A small neoplastic component consistent with B-LBL was detected in BM, along with aggregates of

mastocytes (5–10%), not meeting 2017 WHO criteria for the diagnosis of SM. The patient was negative for KIT D816V on BM aspirate. She received 2 cycles of CVP (cyclophosphamide, vincristine, prednisone) chemotherapy, with no benefit. Subsequently, the treatment with the *FGFR1*-inhibitor Pemigatinib was started and is still ongoing with improvement of the patient's clinical conditions.

Case 8

A 49-year-old man presented with worsening asthenia, marked sweats and unintentional 20 kg weight loss over three months. Blood exams revealed leukocytosis (47,000/mm^3) and mild anemia (Hb 13.8 g/dL) with a high PTL count (520,000/mm^3). The WBC differential showed features of panmyelosis (40% neutrophils, 4% promyelocytes, 8% myelocytes, 8% metamyelocytes) with eosinophilia (3,200/mm^3). CT scan disclosed splenomegaly and diffuse lymphadenopathy. *BCR-ABL1* was negative by FISH on peripheral blood. Histopathological examination of the excised axillary lymph node documented a diffuse proliferation of medium-sized neoplastic cells CD45+, TdT+, CD99+, CD1a+/−, CD10−, CD34−, CD3+, CD5+, CD7+, CD4+, CD8−/+ (rare cells weakly positive), CD56−/+, CD57−, BCL6−/+ (partial, weak expression), BCL2+, CD30−, ALK1−, EMA−, CD79α−, CD20−, MPO−, LMO2−, c-MYC weakly + in 10% of cells, EBER−, Ki67 95%. 5% of myeloid blasts CD34+, CD117+, CD68PGM1− were also identified. Histology was diagnostic of T-LBL. The BM biopsy findings were consistent with MPN with eosinophilia. Cytogenetic analysis on BM aspirate showed a normal 46XY karyotype without any rearrangement. Unfortunately, FISH analyses searching for rearrangements involving *PDGFRA*, *PDGFRB* and *FGFR1* were not performed. The patient received two courses of Hyper-CVAD followed by allo-HSCT. He developed a severe chronic graft versus host disease (cGVHD) treated with steroids and rituximab. At last follow-up (approximately 5 years after allo-HSCT), the patient is in CHR, continuing immunosuppressive therapy with mycophenolate mofetil and extracorporeal photopheresis for cGVHD, mainly involving the skin with scleroderma-like features.

Case 9

A 49-year-old female presented with multiple cervical lymphadenopathy, 2 cm in maximum diameter. Routine blood tests showed a high red blood cell (RBC) count with elevated Hb and HCT levels (Hb 17.8 g/dL; HCT 54%). WBC count was high (31,000/mm^3) with approximately 30% of eosinophils. PTL count (217,000/mm^3) was within normal range. LDH was high (586 U/L). Pathological features of the excised cervical lymph node were consistent with the diagnosis of T-LBL (medium-sized cells with high N/C ratio and dispersed chromatin CD45+; TdT+/−, CD10−/+, CD3+, CD5+, BCL6−, BCL2+/−; CD79α−, LMO2−, Ki67 95%). The BM was hypercellular with trilinear hyperplasia and marked eosinophilia. Cytogenetic studies revealed a translocation involving chromosome 8 short arm and chromosome 13 long arm, t(8;13)(p11;q12) in all analyzed metaphases, compatible with *ZMYM2-FGFR1* rearrangement. Two cycles of hyper-CVAD chemotherapy regimen were administered, with CHR. Due to relapse of disease, the patient received high dose cytarabine (3 g/m^2) as de-bulking therapy, followed by conditioning regimen with busulfan and cyclophosphamide and autologous HSCT. Then, the patient started maintenance therapy with anti-CD52 antibody alemtuzumab, but the disease rapidly progressed and the patient died in 2 months.

Case 10

An asymptomatic 51-year-old woman presented with neutrophilic leukocytosis (WBC 40,000/mm^3), thrombocytopenia (43,000/mm^3) and mild eosinophilia (1,800/mm^3). Hemoglobin was slightly below normal value (Hb 12 g/dL). Flow cytometry on peripheral blood revealed an aberrant T-cell population (7.89% upon all nucleated cells) CD7+, sCD3−, CD4−/+, CD8+, CD16−, CD56+, CD5+, CD2+, cyCD3+. BM biopsy was consistent with MPN/MDS with a mild increase (7%) of immature CD34+ cells. Because of dry tap,

molecular and cytogenetic analyses were not performed on BM aspirate. JAK2 V617F and MPL mutations as well as FISH for *BCR/ABL1* fusion gene were negative on peripheral blood, whereas karyotype documented the presence of four clones: one with t(8;22)(p11;q11); one with trisomy of chromosome 19 plus t(8;22)(p11;q11); one with 48 chromosomes including supernumerary derivative chromosome 22 (der(22)) plus t(8;22)(p11;q11) and trisomy 19; one normal XX female clone. These results were compatible with the presence of *BCR-FGFR1* fusion gene. The patient was started on cytoreductive treatment with hydroxyurea. Two months later, the clinical conditions worsened with the appearance of diffuse lymphadenopathy and splenomegaly (19 cm). The histology of the excised cervical lymph node was consistent with T-LBL showing medium-sized cells with high N/C ratio and dispersed chromatin TdT−/+, CD10 + in some cells, CD34+ in 20%, CD117−, CD45+, CD2+, CD3+, CD5+, CD7+, CD4+, CD8 weak expression in 15%, BCL2+, BCL6 partially and weakly +, PAX5−, CD79α−, CD20−, CD30−, MPO−, CD56−, LMO2+/− (moderate and partial expression), Ki67 > 90%; 10−15% of myeloid blasts MPO+, CD117+, CD34+, CD68PGM1− admixed with T-LBL were observed. Hyper-CVAD chemotherapy regimen was started with only a transient initial response, followed by worsening of the clinical conditions and laboratory findings with leukocytosis and peripheral blasts. The FLA scheme (fludarabine plus high-dose cytarabine) was started, but the patient’s conditions deteriorated and she expired shortly after.

Case 11

A 58-year-old man presented with asthenia, serotinous fever and unintentional weight loss. Laboratory tests disclosed anemia (Hb 10.6 g/dL), low PTL count (50,000/mm^3) and high WBC count (72,830/mm^3) with 34% neutrophils, 5% lymphocytes and 43% eosinophils. Peripheral blood smear showed erythroblasts and dacryocytes along with marked eosinophilia. Multiple palpable enlarged lymph nodes and splenomegaly were present. CT scan confirmed diffuse superficial and profound lymphadenopathy. The histology of the excised lymph node was consistent with T-LBL: CD3+, TdT+, CD1a+, CD4+, CD8+, CD79*α* −, LMO2−, CD34−, CD30 (rare cells). BM biopsy showed hypercellularity (90%) with prevalence of eosinophils. No BM fibrosis was present. JAK2 V617F, calreticulin and MPL mutations as well as FISH for *BCR/ABL1* fusion gene were negative on peripheral blood, whereas *FIP1L1-PDGFRA* fusion gene was detected. Due to progressive increase in eosinophil count (30,000/mm^3) and severe anemia (Hb 9g/dL) requiring transfusion, imatinib (100 mg) was started and it is still ongoing, with rapid eosinophil count decrease (170/mm^3) and reduction of lymphadenopathy.
